# ICU-Associated Costs during the Fourth Wave of the COVID-19 Pandemic in a Tertiary Hospital in a Low-Vaccinated Eastern European Country

**DOI:** 10.3390/ijerph19031781

**Published:** 2022-02-04

**Authors:** Mihai Popescu, Oana Mara Ştefan, Mihai Ştefan, Liana Văleanu, Dana Tomescu

**Affiliations:** 1 Department of Anaesthesia and Intensive Care, Fundeni Clinical Institute, “Carol Davila” University of Medicine and Pharmacy, 022328 Bucharest, Romania; danatomescu@gmail.com; 2 Department of Anaesthesia and Intensive Care, Fundeni Clinical Institute, 022328 Bucharest, Romania; marastefan125@gmail.com; 3 Department of Anaesthesia and Intensive Care, “C. C. Iliescu” Emergency Institute for Cardiovascular Disease, 022328 Buchares, Romania; mihaisteph@yahoo.com (M.Ş.); liana.valeanu@yahoo.com (L.V.)

**Keywords:** COVID-19, healthcare-associated costs, intensive care unit, medication costs

## Abstract

The COVID-19 pandemic has been associated with a tremendous financial and social impact. The pressure on healthcare systems worldwide has increased with each pandemic wave. The present study assesses the impact of the COVID-19 pandemic on healthcare-derived costs of critically ill patients during the fourth wave of the COVID-19 pandemic in a tertiary hospital in Romania. We prospectively included patients admitted to a single-centre intensive care unit (ICU) during the fourth wave of the COVID-19 pandemic. Median daily costs were calculated from financial records and divided in three groups: administrative costs, treatment costs and investigation costs. These were then compared to two retrospective cohorts of non-COVID-19 patients admitted to the same ICU during the same time interval in 2020 and 2019. Demographic data and the management of SARS-CoV-2 infection and of associated organ dysfunctions were recorded to identify risk factors for higher costs. Our results show that the COVID-19 pandemic has been associated with a 70.8% increase in total costs compared to previous years. This increase was mainly determined by an increase in medication and medical-device-related costs. We identified the following as risk factors for increased costs: higher degrees of lung involvement, severity of respiratory dysfunction, need for renal replacement therapy and the use of antiviral or immunomodulatory therapy. Costs were higher in patients who had a shorter duration of hospitalization. In conclusion, the COVID-19 pandemic is associated with increased costs for patients, and rapid measures need to be taken to ensure adequate financial support during future pandemic waves, especially in developing countries.

## 1. Introduction

The COVID-19 (coronavirus disease-19) pandemic that started at the end of 2019 has become the most prominent medical problem worldwide. After the outbreak in the Wuhan region of China [1,2], Europe was struck second, with northern Italy reporting the highest number of critically ill patients [3]. In order to decrease the spread of this novel coronavirus, most European countries imposed lockdown measures with immense social [4], economic [5] and healthcare consequences [6]. As an emergency measure, the European Union, as well as individual countries, begun to develop crisis management plans in order to minimize the overall impact, with various results [7], and intensive care unit (ICU) preparedness began to be the first line of focus, especially for severe forms of the disease [8]. 

In the medical setting, the COVID-19 pandemic had a significant detrimental effect due to an increase in hospital admissions that led to overwhelming of the medical system and insufficient hospital and ICU beds, as well as a lack of sufficient medical personnel and an increase in hospitalization costs [9,10,11]. The incidence of hospital admissions for SARS-CoV-2 (severe acute respiratory syndrome coronavirus-2) infection varies among geographic regions, but up to one quarter of patients needed hospital care, and up to 5% required intensive care measures, with older patients having the highest risk [12,13]. The COVID-19 pandemic had a more significant impact on healthcare systems in developing countries. This led to a shortage of drugs used in the treatment of SARS-CoV-2-infected patients and an increased mortality compared to well-developed countries [14]. Recent studies have demonstrated that the higher COVID-19 mortality observed in developing countries is closely related to the number of ICU beds, medical staff availability and healthcare resources [15].

In Romania, a developing eastern European country, the first wave of COVID-19 was manageable due to the lockdown imposed by national authorities [16,17]. Subsequent outbreaks of the pandemic were more severe with a higher number of patients requiring hospital care and ICU admission [18]. During the fourth wave in the autumn of 2021, the incidence of SARS-CoV-2 infection was around 15 daily new cases per 1000 inhabitants with 20% of infections needing hospitalisation [19]. In the population of around 20 million people living in Romania, the number of hospitalized patients with COVID-19 rapidly reached a peak at the beginning of the fourth wave, and the maximum number of ICU beds designated for SARS-CoV-2-infected patients were fully occupied at the beginning of October [19]. As the medical system in Romania has long been underfinanced, with only 4% of the gross domestic product attributed to healthcare compared with the European average of 7.1% [20], this led to a shortage of necessary drugs, medical equipment and specialized medical care. Hence, in order to improve patient’s outcome, hospital must provide adequate financial support, especially for critically ill patients [21,22].

To date there are limited data on the economic effects of the COVID-19 pandemic on healthcare systems in developing countries. Although similar reports from Western Europe and North America exist, they may not reflect the financial situation in other poorer regions or their impact on national budgets. Additionally, there are insufficient data to compare COVID-19-associated costs with those of non-COVID-19 patients. The aim of this study was to assess the healthcare-associated costs of SARS-CoV-2-infected patients and identify factors associated with higher costs. Secondary endpoints were to compare COVID-19-derived costs with those of non-COVID-19 patients from a general ICU in a single tertiary hospital in Romania.

## 2. Materials and Methods

The ethical approval for the present study was provided by the Ethical Committee of Fundeni Clinical Institute, Bucharest, Romania (approval number 21195/2021). All patients signed an informed consent for participating in observational studies at the time of hospital admission. 

*Patient inclusion*. In the present study, we prospectively included consecutive patients diagnosed with COVID-19 (COVID-19 group) who were admitted in the general ICU of Fundeni Clinical Institute, Bucharest, Romania, between 1 September and 31 October 2021. During this time, the ICU functioned as a critically ill COVID-19 ICU, so only patients diagnosed with SARS-CoV-2 infection were admitted. The COVID-19 group was compared with two retrospective groups: the 2020 group and 2019 group. The 2020 group and the 2019 group consisted of all consecutive patients who were admitted in the same ICU between 1 September–31 October 2020, and 1 September–31 October 2019, respectively. All patients in the 2020 group were non-COVID-19 patients as the ICU functioned as a non-COVID-19 unit during that time. Additionally, all patients in the 2019 group were non-COVID-19 as it was before the start of the pandemic. 

*Patient exclusion.* The exclusion criteria were patients admitted in the ICU following planned or emergency surgery and length of ICU admission shorter than 24 h.

The diagnosis of COVID-19 was performed by either rapid antigen testing or RT-PCR (reverse transcription–polymerase chain reaction) for SARS-CoV-2 at the time of hospital admission, and the infection was reconfirmed by RT-PCR at ICU admission. Patients were treated by the attending physician in accordance with national guidelines published in the Official Gazette number 978/2021. 

The following data were recorded for the three groups: age, sex, ICU length of stay (ICU LoS), hospital length of stay (Hospital LoS) and outcome. Severity of illness at the time of ICU admission was assessed using two international validated scores for ICU patients: the SOFA (Sequential Organ Failure Assessment) score [23] and APACHE II (Acute Physiology and Chronic Health Evaluation) score [24]. All costs reimbursed by national authorities were calculated using the financial records of each patient and divided into three groups: administrative costs (food and utilities), paraclinical costs (radiology and laboratory tests) and treatment costs (medication and medical-device-related disposables). Of note, personnel costs were not included in administrative costs. This is because personnel costs are not reported in the healthcare system in Romania, and so they are not reimbursed by national authorities. Additionally, as medical device depreciation is not reimbursed in Romania, device-related costs refer only to disposables and not to the device itself. The total cost per patient was calculated by the mathematical sum of the above costs. The mean daily total cost per patient was calculated as follows: the total cost of each patient was divided by the number of hospital days of that patient. Costs were reported in national currency, the Romanian leu, and all sums were converted to euros (EUR) using the average exchange rate reported monthly by the National Bank of Romania for the specified time [25].

In order to assess factors associated with higher costs for patients with COVID-19 the following data were collected: age, sex, status of vaccination, severity of pulmonary involvement as assessed by computer tomography at the time of ICU admission, the number of days between first symptoms of COVID-19 and ICU admission, APACHE II and SOFA scores and P/F ratio (arterial partial pressure of oxygen / fraction of inspired oxygen) at the time of ICU admission, number of days on HFNC (high-flow nasal canula), number of days on NIV (non-invasive mechanical ventilation), number of days on IMV (invasive mechanical ventilation), need for renal replacement therapy, need for invasive cardiac output monitoring, antiviral therapy, immunomodulatory therapy (anti-interleukine-6 drugs), number of ICU days, number of hospital days and outcome (defined as 28-day survival). Factors were selected based on previous studies performed on critically ill ICU patients [26,27] as well as factors specific to COVID-19 treatment (use of antiviral and immunomodulatory drugs). 

*Statistical analysis*. Statistical analyses were performed using SPSS 19.0 (SPSS Inc^®^, Chicago, IL, USA). Data distribution was examined to ensure the proper statistical examination by the Shapiro–Wilk test. If the data followed a normal distribution, they were reported as mean ± standard deviation of the mean and as median (min, max) if they did not follow a normal distribution. The comparison of the three groups was performed using ANOVA if the data were normally distributed or Kruskal–Wallis test if non-normally distributed. Inter-group variability was further assessed by post hoc analysis using Bonferroni test. Increased costs were defined as daily total costs higher than the median, EUR 598.4. Categorical variables were analysed with Chi-square test, and quantitative data were analysed with independent samples *t*-test or Mann–Whitney test when the analysed data did not follow a normal distribution. The correlation between hospital LoS and mean daily costs was performed using Spearman correlation. Survival was assessed using Kaplan–Meier analysis, and the *p* value was derived using log-rank test. All *p* values are two-tailed, and a *p* value of less than 0.05 was considered statistically significant.

## 3. Results

One-hundred and twenty-five patients were included in the final analysis: 36 patients in the COVID-19 group, 43 patients in the 2020 group and 46 patients in the 2019 group. There was no difference in age, ICU LoS or hospital LoS among the three groups. Data are presented in Table 1. Patients in the COVID-19 group had a significantly lower SOFA score compared to the 2020 group (4.9 ± 2.9 vs. 9.5 ± 4.1, *p* < 0.01) and 2019 group (4.9 ± 2.9 vs. 9.1 ± 3.9, *p* < 0.01) with no difference in SOFA score between the 2019 group and 2020 group (*p* = 0.95). The APACHE II score was also noted to be significantly lower in the COVID-19 group compared to the 2020 group (14.7 ± 7.5 vs. 24.2 ± 10.0, *p* < 0.01) and the 2019 group (14.7 ± 7.5 vs. 22.6 ± 6.0, *p* < 0.01) with no difference in the APACHE II score between the 2019 group and 2020 group (*p* = 0.76). Survival was highest in the COVID-19 group compared to the 2020 and 2019 groups but did not reach statistical significance.

**Figure 1:**
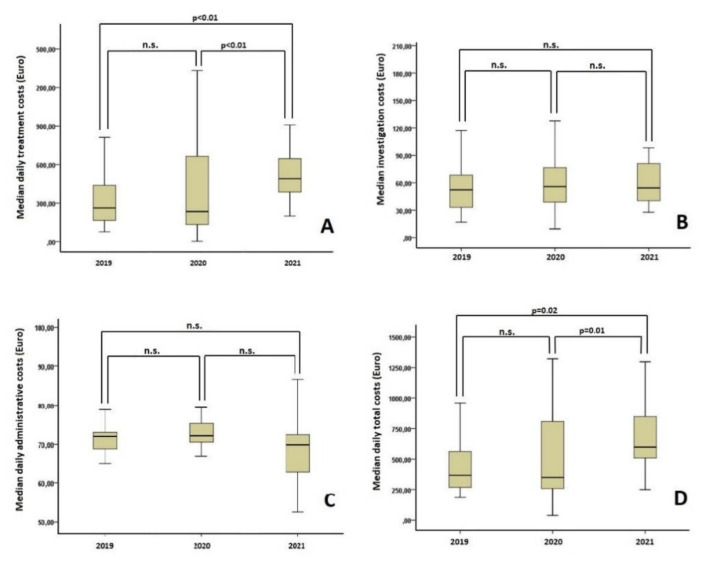
Comparison of costs among the three time cohorts. (**A**). Comparison of treatment costs; (**B**) comparison of investigation costs; (**C**) comparison of administrative costs; (**D**) comparison of total costs. n.s.—non-statistically significant.

Median daily total costs increased by 70.8% in the COVID-19 group compared to the 2020 group (EUR 598.4 [249.1, 1296.7] vs. EUR 350.2 [38.7, 2884.7], *p* = 0.01) and by 63.2% compared to the 2019 group (EUR 598.4 [249.1, 1296.7] vs. EUR 366.6 [186.2, 2624.3], *p* = 0.02). This was due to significantly higher daily treatment costs in the COVID-19 group compared to the 2020 group (EUR 490.4 [199.3, 1095.0] vs. EUR 234.5 [12.8, 2045.3], *p* < 0.01) and the 2019 group (EUR 490.4 [199.3, 1095.0] vs. EUR 263.6 [76.6, 2435.1], *p* < 0.01). No significant difference was observed among the COVID-19 group and the 2020 and 2019 groups regarding administrative and paraclinical daily costs. Additionally, no significant differences were noted in terms of treatment, paraclinical, administrative and total costs between the 2020 and 2019 groups. Data are presented in Table 1 and Figure 1.

**Figure 2:**
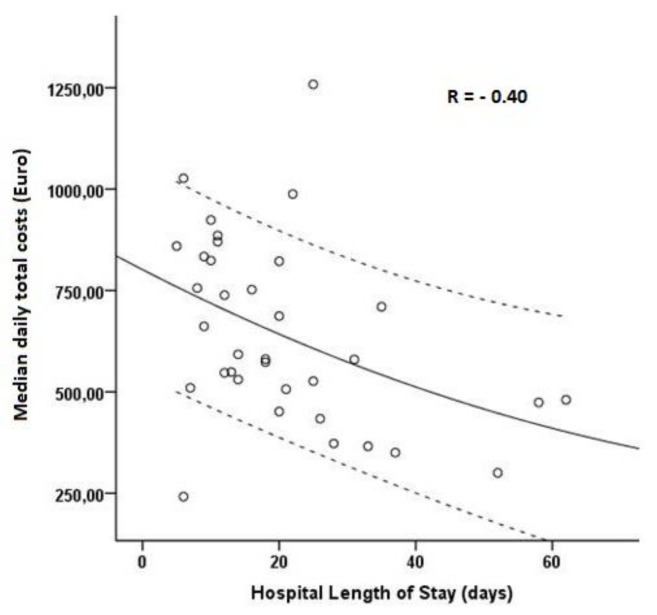
Correlation between total daily costs and hospital length of stay.

The median total cost for COVID-19 patients was EUR 10,319 [1450,31,459]. In the COVID-19 group the factors that strongly correlated with higher total daily costs were degree of lung involvement (*p* < 0.01), lower P/F ratio (*p* < 0.01), need for renal replacement therapy (*p* = 0.02) and treatment with antiviral (*p* = 0.02) or immunomodulatory (*p* < 0.01) drugs. All patients who were treated with immunomodulatory drugs received this therapy within 24 h of ICU admission. We also observed an inverse correlation between the length of hospital stay and median daily total costs (R = −0.40, *p* = 0.01), with patients having the shortest duration of stay having the highest daily total costs—Figure 2. Data are presented in Table 2.

## 4. Discussion

COVID-19 has become the largest medical challenge of recent times with tremendous effects on national economies, labour, access to medical facilities and psychological wellbeing. Although these effects have been well documented in large-scale studies from different geographical and cultural areas [28], the impact of the COVID-19 pandemic on the financial resources of public healthcare systems has not been fully described, especially in low-income and developing countries. 

In our study we report a median daily cost for COVID-19 patients of EUR 598.4 and a median hospitalization cost of EUR 10,319. Before comparing these costs with those reported by other studies, most of which have been published in developed or high-income countries, several factors must be considered. First, medical systems and cost reporting varies among countries. The healthcare system in Romania only reimburses direct costs, that is, medication, medical imaging, laboratory tests, device-related disposables, food and utilities. Personnel costs and medical device maintenance are covered by the hospital budgets, which are DRG (diagnosis-related group) based. Only one-third of Romania’s population pays medical insurance as a fixed percentage from their income, while the rest is covered by the national budget as social security [29]. This approach has led to a medical system that is severely underfinanced [30]. 

In an estimation of direct medical costs of hospitalized COVID-19 patients in Saudi Arabia, Khan et al. [31] reported a mean daily cost per ICU patient of USD 7810 and of USD 11,215 for patients requiring mechanical ventilation. These daily costs were almost 20 times higher than the costs reported by our study. However, the authors did not provide any details related to the type of costs included so that we cannot say if these higher costs are attributed to a higher medication cost or to the addition of supplemental costs such as personnel costs. In a similar study published by Oksuz et al. [32], the authors reported the same treatment protocol as that applied in our cohort with a mean medication cost of ICU patients of USD 207.9 per day and a mean total patient cost of USD 3461.1. The difference between this study and ours can be explained by the added cost of immunomodulatory therapy and medical-device-related costs that were not reported in the Turkish cohort, although the same antiviral therapy, a major determinant of cost increasement in our study, was used in both the Romanian and Turkish cohorts. Other studies from the United States and Western Europe show similar median medication costs as those reported by our data [33,34] but report a much higher daily patient cost. One of the major differences observed between our study and others from more well-developed countries is that personnel and administrative costs have a major impact on the overall budget, and patients’ costs vary tremendously if these costs are included. For example, in one study by Carrera-Hueso et al. [33] the mean cost for COVID-19 patients requiring ICU was EUR 280.956; however, 97.3% of this was attributed to hospital care and management, while only 0.6% of the total costs were drug-related costs. Although the medication costs were similar to ours, they reported 20-times higher costs for medical imaging and laboratory tests. 

Our results show that the management of COVID-19 is more expensive than that of non-COVID-19 critically ill patients, with treatment and medical-device disposables having the biggest impact. Previous studies have demonstrated that patient costs are related to the severity of illness, ICU length of stay, need for mechanical ventilation and renal replacement therapy [22]. However, ICU costs are specific to patient populations and are dependent on specific disease treatments. Our data demonstrate that the overall increase in patient costs is determined by an increase in treatment costs, as most patients in our cohort required both antiviral and immunomodulatory therapy. As previously mentioned, many healthcare systems in developing countries are underfinanced and, as such, healthcare budgets are predicted based on expenses during the previous years. As the treatment of critically ill COVID-19 patients is significantly more expensive than the average treatment of non-COVID-19 patients, a correction must be made in order to ensure proper financing for ICUs and acute care wards.

In the COVID-19 cohort, we reported a mortality of 47.3%. This is similar to the median mortality of ICU patients reported by other studies [35]. However, our results show that COVID-related mortality was lower than that of non-COVID-19 patients from the previous two years, but this did not reach statistical significance. This trend towards a lower mortality may be attributed to the fact that most patients presented with a single organ dysfunction and were admitted to our ICU due to severe respiratory failure. This is demonstrated by the lower mean SOFA and APACHE II scores in 2021 compared with 2020 and 2019. Lower severity scores have also been reported by other studies that noted a respiratory SOFA sub-score as the main determinant [36,37] of severity. Additionally, there is a far inferior correlation between predicted mortality based on either SOFA or APACHE II scores and the observed mortality [38]. 

In our cohort, a higher total daily cost was associated with higher degrees of lung involvement, severity of respiratory dysfunction, need for renal replacement therapy and the use of antiviral or immunomodulatory therapy. Di Fusco et al. [39] argued that invasive mechanical ventilation was associated with higher patient costs; however, our study showed that the severity of lung involvement and respiratory dysfunction, as demonstrated by the P/F ratio, are more closely related to higher costs regardless of the respiratory therapy used. This is because the cost for mechanical ventilation disposables, either high-flow nasal cannula or ventilation circuits for invasive or non-invasive mechanical ventilation, are similar. In our study, acute kidney injury was the most common organ dysfunction after respiratory failure in COVID-19 patients, and about one in four patients required renal replacement therapy. This is in accordance with large-scale studies conducted in the United States that showed a similar incidence of renal failure [40]. However, this is almost half of the incidence reported by previous studies in critically ill non-COVID-19 patients [41]. Although renal replacement therapy may be a substantial contributor to higher daily costs of COVID-19 patients, it may not be responsible for the cost difference between COVID and non-COVID patients. Based on our data, patients with a shorter ICU LoS have higher daily costs. This is in contradiction with previously published data from non-COVID patients that demonstrate a correlation between ICU LoS and costs [42,43]. One of the main reasons for our results is that the use of immunomodulatory therapy was associated with increased costs. All patients received this treatment in the first 24 h after ICU admission for a total of three consecutive days. Patients with a shorter ICU LoS received the same antiviral and immunomodulatory therapy as those with longer admission times. However, when divided by the number of hospitalization days, the median daily costs are higher due to similar total costs divided by a higher number of hospitalization days.

The COVID-19 pandemic has had a major impact on the Romanian national healthcare system. The median daily costs have increased by approximately 70% compared to previous years and, as the national medical system has been chronically underfinanced, this has led to the inability to cover hospitalization costs during the peak of the fourth wave. On the other hand, we have observed a decrease in the number of patients that were treated in our ICU since the start of the pandemic compared to pre-pandemic years. This was due to epidemiologic measures designed to decrease the risk of cross-contamination between COVID and non-COVID patients, as well as partial restrictions on admission polities applied by the Ministry of Health. Thirdly, in January 2021, the vaccination campaign started, and, in the first few months, Romania was among the first countries with the highest percentage of the population being vaccinated [44]. However, due to the inability of public authorities to promote vaccination and the overwhelming number of fake news regarding major side effects of vaccination, the number of adults willing to be vaccinated against SARS-CoV-2 infection began to decline and, at the beginning of September 2021, only 26.8% of the population aged 16 and above was vaccinated [45,46,47]. This low percentage of vaccination coincided with the beginning of the fourth wave of the pandemic. In order to minimize the financial and medical consequences of future pandemic waves, vaccination against SARS-CoV-2 has to increase significantly in order to decrease the number of severely affected patients who require hospital and ICU admission [48]. 

Our study has some important limitations. First, there is no universally accepted protocol for the treatment of COVID-19 patients, and costs may vary among countries depending on local treatment guidelines as previously mentioned. However, the COVID-19 management developed by our national authorities follows the general guidelines and recommendations developed by the World Health Organisation as well as other international recognized medical societies. The management of severe organ failure and the use of antiviral or immunomodulatory therapy applied to our patients is in accordance with that of the Surviving Sepsis Campaign [49], and so the derived costs are comparable between countries that adhere to these guidelines. Second, as reported healthcare costs may differ from one healthcare system to another, a comparison is hard to make, so our observations regarding total costs may not apply to other countries or healthcare systems, especially those of well-developed countries. As most countries adhere to the Surviving Sepsis Campaign as previously mentioned, a comparison between medication costs can be made. Third, antiviral or immunomodulatory treatment may not be universally available, including in some hospitals from Romania, and the overall costs may vary depending on medication availability. However, regardless of its limitations, our study demonstrates some key aspects in health economics and public health measures regarding healthcare-associated costs of COVID-19 patients compared to non-COVID patients. If in Western Europe and other developed countries there is a lack of sufficient ICU beds and specialized medical personnel, as well as the burn-out of healthcare workers, which has proved to be the main problem in dealing with the current pandemic, in underdeveloped or developing countries, ensuring adequate budgets to cover healthcare-associated costs also represents a crucial issue. Considering this, national authorities should urgently ensure appropriate funding for the medical treatment of COVID-19 critically ill patients as future waves of the pandemic are expected.

## 5. Conclusions

In conclusion, our data show that the treatment of critically ill COVID-19 patients, despite having lower severity scores, is associated with significantly higher daily costs due to an increase in treatment costs when compared to non-COVID patients. Median daily costs were higher in patients with a shorter hospitalization. Severity of respiratory failure and the need for renal replacement therapy were associated with higher costs. This is a problem in underfinanced healthcare systems especially; therefore, to avoid the inability to treat critically ill patients, adequate financial support based on specific costs for the management of these patients should be implemented by national authorities. Future studies should focus on developing prediction models for the number of critically ill COVID-19 patients in order to better match budgetary demands to the number of patients.

## Figures and Tables

**Table 1:** Comparison of demographic data and mean daily costs among the three time cohorts.

Variable	Year 2021 (*n* = 36)	Year 2020 (*n* = 43)	Year 2019 (*n* = 46)	*p* Value ^	*p* Value 2021 vs. 2020	*p* Value 2021 vs. 2019
Age (years)	62.0 ± 15.9	60.4 ± 18.7	62.0 ± 15.9	0.73	0.91	0.92
APACHE II Score	14.7 ± 7.5	24.2 ± 10.0	22.6 ± 6.0	<0.01 *	<0.01 *	<0.01 *
SOFA Score	4.9 ± 2.9	9.5 ± 4.1	9.1 ± 3.9	<0.01 *	<0.01 *	<0.01 *
ICU LoS (days)	14.1 ± 8.1	11.4 ± 9.5	11.0 ± 5.6	0.17		
Hospital LoS (days)	20.1 ± 14.0	22.3 ± 15.9	19.7 ± 11.3	0.64		
Survival (%)	52.7%	37.2%	43%	0.27		
Median daily treatment cost (euro)	490.4 [199.3, 1095.0]	234.5 [12.8, 2045.3]	263.6 [76.6, 2435.1]	<0.01 *	<0.01 *	<0.01 *
Median daily investigation costs (euro)	54.3 [27.7, 483.0]	55.9 [9.5, 401.1]	52.2 [16.9, 765.2]	0.07		
Median daily administrative costs (euro)	69.8 [25.8, 174.4]	72.2 [6.6, 689.8]	71.9 [34.9, 720.1]	0.09		
Median daily total costs (euro)	598.4 [249.1, 1296.7]	350.2 [38.7, 2884.7]	366.6 [186.2, 2624.3]	<0.01 *	0.01 *	0.02 *

Legend: APACHE II score—Acute Physiology and Chronic Health Evaluation score; SOFA Score—Sequential Organ Failure Assessment score; ICU—intensive care unit; LoS—length of stay. *—indicates statistical significance; ^—statistical comparison among the three groups.

**Table 2:** Risk factors for increased costs in the COVID-19 cohort.

Variable	Value	*p* Value
Age (years)	62.0 ± 15.9	0.88
Vaccinated (%)	16.7% (*n* = 6)	
Lung involvement (%)	60 [30, 90]	<0.01 *
APACHE II Score	14.7 ± 7.5	0.76
SOFA Score	4.9 ± 2.9	0.49
Days between first symptoms and ICU admission	7.1 ± 4.2	0.27
P/F ratio	128 ± 50	<0.01 *
Need for invasive mechanical ventilation Days on HFNC	55.6% (*n* = 20) 1.5 [0, 8]	0.38 0.29
Days on NIV	3.0 [0, 15]	0.64
Days on IMV	1 [0, 33]	0.24
Need for RRT	22.2% (*n* = 8)	0.02 *
Need for invasive cardiac output monitoring	33.3% (*n* = 12)	0.22
Antiviral treatment	88.9% (*n* = 32)	0.02 *
Immunomodulatory treatment	36.1% (*n* = 13)	<0.01 *
ICU LoS	14.1 ± 8.1	0.81
Hospital LOS	20.1 ± 14.0	0.01 *
Survival (%)	52.7% (*n* = 19)	0.26

Legend: APACHE II score—Acute Physiology and Chronic Health Evaluation score; SOFA Score—Sequential Organ Failure Assessment score; ICU—intensive care unit; P/F ratio—arterial partial pressure of oxygen/fraction of inspired oxygen; HFNC—high-flow nasal canula; NIV—non-invasive mechanical ventilation; IMV—invasive mechanical ventilation; RRT—renal replacement therapy; LoS—length of stay; *—indicates statistical significance.

## Data Availability

Data can be requested from the corresponding author.
